# Ultrasonic Cavitation Erosion Behavior of AlCoCr*_x_*CuFe High Entropy Alloy Coatings Synthesized by Laser Cladding

**DOI:** 10.3390/ma13184067

**Published:** 2020-09-13

**Authors:** Danqing Yin, Guangbing Liang, Shuai Fan, Shanxin Li

**Affiliations:** School of Materials Science and Engineering, Henan University of Science and Technology, Luoyang 471003, China; gbliang1995@stu.haust.edu.cn (G.L.); f674352798@163.com (S.F.); lx2839053045@126.com (S.L.)

**Keywords:** ultrasonic cavitation erosion, high entropy alloy coatings, AlCoCr*_x_*CuFe, laser cladding

## Abstract

Cavitation corrosion resistant coatings are an excellent solution to the cavitation corrosion problem. High entropy alloys provide a new possibility for cavitation resistant coatings due to their excellent comprehensive performance. Laser cladding was employed to synthesize AlCoCr*_x_*CuFe (*x* represents the Cr concentration, *x* = 0.5, 1.0, 1.5, 2.0) high entropy alloy coatings (HECs) on AISI 304 steel. The phase transformation, microstructure, micro-mechanical properties, and cavitation erosion performance of HECs were studied. Results showed that AlCoCr*_x_*CuFe HECs were composed of BCC and FCC duplex phase. The microstructure of HECs showed a typical dendritic structure. The composition segregation of interdendrite structures was observed. Cavitation erosion resistance represented by 20 h volume loss was decreased with the increase in Cr content. AlCoCr*_x_*CuFe HECs with the lowest chromium content (AlCoCr_0.5_CuFe) showed the best cavitation erosion resistance among all samples. The cavitation resistance of AlCoCr*_x_*CuFe HECs has good correlation with the mechanical parameter $H_{n}{}^{3}$/$E_{r}{}^{2}\;$($H_{n}$ is nanohardness, $E_{r}$ is elastic modulus) and phase formation parameter δ (δ is atomic radius difference). The surface after 20 h of cavitation erosion testing exposed the dendritic structure of BCC phase, which was caused by the destruction of the interdendrite structure by cavitation impact.

## 1. Introduction

Cavitation is a common phenomenon in ship propellers, rudder blades, turbine impellers, pumps, and other hydraulic machinery [1]. Water flow will cause serious vibration, noise, and material damage in hydraulic machinery during operation due to cavitation erosion. In the flow system, the static pressure at any position lower than the vapor pressure of the liquid will cause cavitation in the water. When the bubble pressure returns to higher than the steam pressure, the generated steam bubbles will be transported by the fluid and collapse accordingly. If bubbles continue to burst near the solid boundary, it will lead to surface material damage and component failure [2,3,4].

The coatings can not only provide effective protection for the parts prone to cavitation damage, but also save valuable materials. In the research of cavitation erosion resistant materials, different technologies have been used to synthesize cavitation erosion resistant materials or cavitation erosion resistant coatings. Chiu K Y et al. [5] reported Ni–Ti coatings were synthesized by laser cladding using relatively economical Ni–Ti strips. The results show that Ni–Ti coatings have the characteristics of high hardness, high pressure mark recovery rate (unloading recovery displacement to maximum displacement), no cracks and no pores, which lead to the high erosion resistance of Ni–Ti coatings. Singh R et al. [6] has synthesized a novel coating named Stellite 6 on 13Cr-4Ni stainless steel with laser cladding. Correlation between the laser power and cavitation erosion resistance of the coatings has also been studied. In their work, a high velocity oxygen fuel (HVOF) spraying process was used by Taillon G et al. [7] to synthesize WC-CoCr and Cr3C2-NiCr HVOF metal-ceramic coatings. These coatings showed obviously lower erosion rates than the substrate. In addition, Wang Y et al. [8] concluded that the mean erosion depth of CoMoCrSi coatings synthesized by atmospheric plasma spraying (APS) was significantly reduced.

High entropy alloys (HEAs) were first proposed by Jien-Wei Yeh of Taiwan Tsinghua University in 1995 on the basis of research on amorphous alloys [9]. HEAs have attracted more and more attention due to its multi-component solid solution structure and excellent comprehensive performance [10]. Excellent properties of HEAs have been observed. In most HEAs, very high strength and high plasticity [11] are commonly observed. Some HEAs showed high corrosion resistance [12], low temperature resistance [13], and high temperature oxidation resistance [14]. High fatigue resistance and high wear resistance were observed in the research of Hemphill M A et al. [15] and Zhang H X et al. [16], respectively. Therefore, HEAs will have a wide application prospect in cavitation corrosion resistant materials [17]. HEAs, previously known as multiple principal element alloys, are generally formed by five or more principal elements. The molar content of HEAs constituent elements is equal or approximate. Now, broadening design scope has allowed the content of the principal element in HEAs to vary between 5% and 35% (atomic fraction). Amongst all of the characteristics, the high entropy effect is the most essential one. The reason is because only a single-phase or duplex-phase solid solution can be formed in most of the HEAs believed to be governed by the high entropy effect [18].

The atoms of each kind of element in the HEAs occupy the lattice position of the crystal randomly and each atom is surrounded by the atoms of other elements. However, due to different atomic sizes and different properties, severe lattice distortion will occur in solid solutions [19,20]. Formation of the second phase will be reduced due to the lower diffusion rate of atoms in HEAs caused by severe lattice distortion. Another unique effect is called the “cocktail effect”, which means that there will be a complex interaction between the principal elements that leads to new characteristics in HEAs [21,22].

In recent years, the research into HEAs has received extensive attention, however, only a few have concentrated on their cavitation erosion resistance. Wu C L et al. [23] prepared FeCoCrAlNiTi*_x_* high entropy alloy cavitation erosion resistant coatings using laser cladding. They studied the influences of Ti content on the cavitation erosion behavior of their HEAs and concluded that with the increase in Ti content, FeCoCrAlNiTi_2_ showed the best cavitation resistance in distilled water, which was due to the hard intermetallic phases Ti_2_Ni and NiAl in FeCoCrAlNiTi_2_. Nair R B et al. [24] investigated the difference of cavitation erosion resistance between Al_0.1_CoCrFeNi high entropy alloys and 316 L stainless steel. Results show that the cavitation erosion rate of Al_0.1_CoCrFeNi high entropy alloys is about 1/4 that of 316 L stainless steel. In the present work, AlCoCr*_x_*CuFe HECs were prepared on an AISI 304 steel substrate using the laser cladding technique. The effect of Cr content on microstructure, micro-mechanical properties, and cavitation erosion performance was investigated. Effect of the micro-mechanical properties (nanohardness, elastic modulus) and phase formation parameter (mixing entropy, atomic radius difference) were attempted to develop a further understanding of the cavitation erosion resistance of HECs.

## 2. Materials and Methods

### 2.1. Coatings Preparation

The substrate used in the current study was an AISI 304 steel plate. The sample size was 60 mm × 60 mm × 10 mm. Fabrication of AlCoCr*_x_*CuFe HECs was performed using the LDM-6000 type laser machine (Laserline, Australia). The prepared AlCoCr*_x_*CuFe coatings were denoted Cr*_x_* where *x* corresponded to the molar ratio of Cr to each of additional elements multiplied by a factor of ten. The nominal composition of AISI 304 steel in this work is listed in Table 1. Al, Co, Cr, Cu, and Fe powders were used for laser cladding and the powders’ particle size was 150 μm. The laser cladding parameters are shown in Table 2. All powders were spherical powders with a purity higher than 99.9%. The mixed powder was ball milled with a rotation speed of 60 r/min for 8 h under argon protection. Before the experiment, the mixed powder was oven dried at 70 °C for 2 h.

### 2.2. Microstructural Characterization

After the laser cladding process, AlCoCr*_x_*CuFe HECs specimens were sectioned into 10 mm × 10 mm × 10 mm pieces perpendicular to the laser cladding path. The cross section of the specimens was ground with 2000 grit sandpaper, polished to a scratch free mirror surface, and subjected to 10% oxalic acid (beilianchem, 99.5%) solution electrolytic corrosion for 30 s. Specimens after oxalic acid electrolytic corrosion surfaces were observed using a scanning electron microscope (SEM) (JEOL JSM-7800F, Tokyo, Japan) with energy dispersive spectroscopy (EDS) (JEOL JSM-7800F, Tokyo, Japan). X-ray diffraction (XRD) (Bruker D8 Advance, Billerica, MA, USA) was used to analyze the phase structure of HECs at a scanning speed of 4° min^−1^, ranging from 20° to 100°. The nanoindentation characteristics of the AlCoCr*_x_*CuFe HECs were studied by a nanoindentation tester (Keysight Nanoindenter G200, Santa Rosa, CA, USA). The measurements were carried out using a Berkovich indenter with the load of 300 mN and loading time of 25 s. Nanoindentation tests were performed for each sample five times.

### 2.3. Cavitation Erosion Test

**Figure 1:**
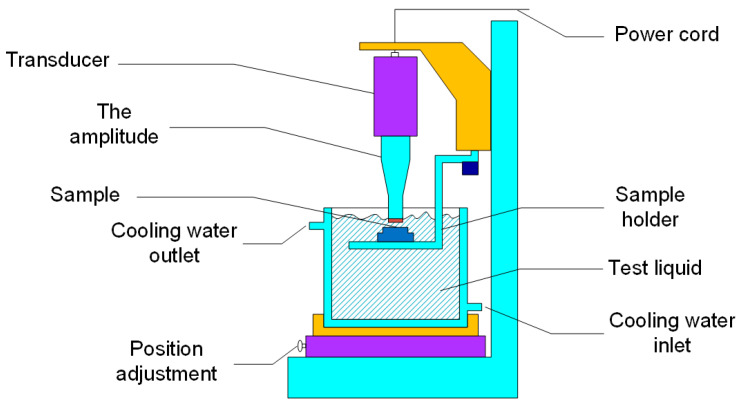
Diagram of the ultrasonic cavitation erosion device.

Ultrasonic cavitation equipment conforming to the ASTM G-32 standard [25] was adopted. The vibration frequency was 20 kHz and the amplitude was 50 μm peak-to-peak. The schematic diagram of the cavitation erosion device used in the current work is shown in Figure 1.

The surface of the laser cladding sample was ground with 2000 grit sand paper before the experiment. During the experiment, the sample was immersed in a container filled with distilled water. The vibrating head was 10 mm under the liquid surface, and the diameter of the vibrating head was 16 mm. The sample surface was 0.5 mm below the vibrating head surface. The cooling system including a beaker and water-cooler kept the water temperature at 25 °C. The accumulated time of the ultrasonic cavitation erosion experiment was 20 h, and mass loss (ML) was measured every hour with a balance with the sensitivity of 0.1 mg. Mass loss (ML) was converted to volume loss (VL) to eliminate the density effect. The surface of AlCoCr*_x_*CuFe HECs after the cavitation erosion test (20 h) was observed with a scanning electron microscope (JEOL JSM-7800F, Tokyo, Japan).

## 3. Results

### 3.1. Phase Formation

**Figure 2:**
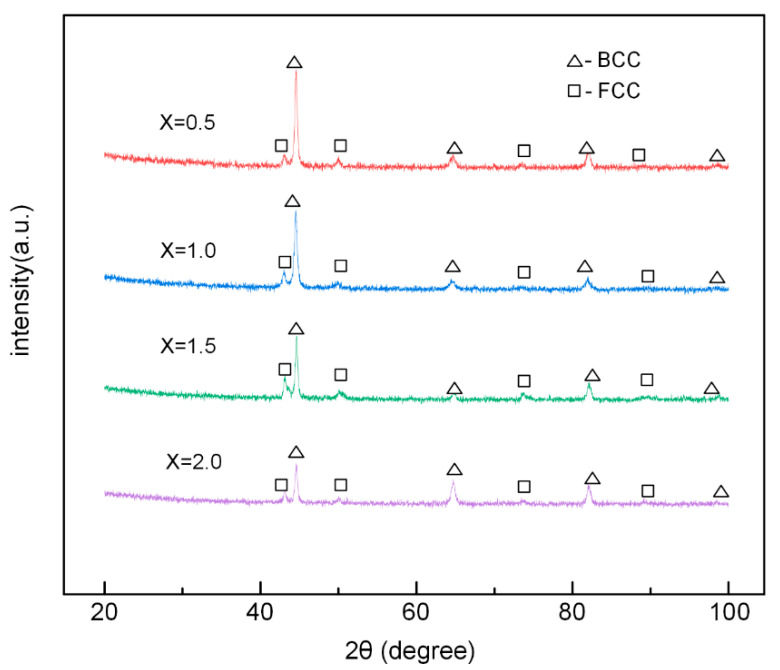
XRD (X-ray diffraction) patterns of AlCoCr*_x_*CuFe HECs.

Phase formation analysis was carried out. Results of the XRD patterns of AlCoCr*_x_*CuFe HECs including different Cr content is shown in Figure 2. As can be clearly seen, Cr05, Cr10, Cr15, and Cr20 HECs all showed FCC and BCC duplex phase structures. According to a previous report by [26] of AlCoCrCuFe HEA, the disordered BCC phase and FCC phase was identified, which was consistent with the results of the current study. The strongest diffraction peak of BCC phase was at about 43°. With the addition of Cr content, the strongest diffraction peak of BCC decreased. As the alloy was composed of two phases and no intermetallic compound phase was obviously formed. It can be judged that with the increase in the Cr element, the content of the BCC phase decreased gradually. In addition, a significantly enhanced BCC peak was generated at 65° due to Cr aggregation between the dendrites at *x* = 2.0 [27].

The mixing entropy ($\Delta S_{\mathrm{mix}}$) of the AlCoCr*_x_*CuFe HECs was positive and mixing enthalpy ($\Delta H_{\mathrm{mix}}$) was negative. The Gibbs free energy of HECs was negative, as shown by the Equation (1), which was beneficial to form the solid solution phase. Related research [28,29] has shown that the formation of the HEAs phase is influenced by properties such as $\Delta S_{\mathrm{mix}}$, $\Delta H_{\mathrm{mix}}$, atomic radius difference (δ), electronegativity difference (Δχ), valence electron concentration (VEC), and Ω. These parameters can be expressed as Equations (2)–(7):

(1) $$\Delta G_{\mathrm{mix}}=\Delta H_{\mathrm{mix}}-T\Delta S_{\mathrm{mix}}$$

(2) $$\Delta S_{\mathrm{mix}}=Rlnn$$

(3) $$\Delta H_{\mathrm{mix}}=\overset{n}{\underset{i=1,i\neq j}{\sum }}4\Delta H_{ij}^{mix}c_{i}c_{j}$$

(4) $$\delta =\sqrt{\overset{n}{\underset{i=1}{\sum }}c_{i}{\left(1-r_{i}/\bar{r}\right)}^{2}}$$

(5) $$\Delta \chi =\sqrt{\overset{n}{\underset{i=1}{\sum }}c_{i}{\left(\chi_{i}-\bar{\chi }\right)}^{2}}$$

(6) $$\mathrm{VEC}=\overset{n}{\underset{i=1}{\sum }}c_{i}{\left(\mathrm{VEC}\right)}_{i}$$

(7) $$\Omega \left(T\right)=\frac{T_{m}\Delta S_{\mathrm{mix}}}{\left|\Delta H_{\mathrm{mix}}\right|}$$

The meaning of the parameters in the equation is as follows:

$\Delta G_{\mathrm{mix}}$—Gibbs free energy.

*R* = 8.314 J/(mol·K)—gas constant.

*n*—the number of element types.

$\Delta H_{ij}^{mix}$—the mixing enthalpy of the *i* principal element and the *j* principal element in regular solution.

$c_{i}$ and $c_{j}$—the atomic contents of the *i* principal element or the *i* principal element. 

$\bar{r}$—the average radius of the alloy element atoms.

$r_{i}$—the radius of the *i* element. 

$\bar{\chi }$—the average electronegativity of the elements.

$\chi_{i}$—the electronegativity of the *i* element.

$T_{m}$—the melting point of the alloy.

The calculation results of the parameters of the laser cladding AlCoCr*_x_*CuFe HECs in this study such as the $\Delta S_{\mathrm{mix}}$, $\Delta H_{\mathrm{mix}}$, δ, Δχ, VEC, and Ω are shown in the Table 3. The physical chemical/thermodynamic parameters of the alloy elements used in this study are shown in Table 4 and Table 5.

Zhang Yong et al. [31] believed that the formation range of the solid solution phase was δ < 6.5%, −15 kJ/mol < $\Delta S_{\mathrm{mix}}$ < 5 kJ/mol, 12 J/(K·mol) < $\Delta H_{\mathrm{mix}}$< 17.5 J/(K·mol). Guo et al. [32] concluded that FCC solid solutions would be relatively stable when VEC ≥ 8.6, and BCC solid solutions will be relatively stable when VEC < 6.87. Moreover, Zhang Yong et al. [33] indicated that the $\Delta H_{\mathrm{mix}}$ and *T*$\Delta S_{\mathrm{mix}}$ can both affect the $\Delta G_{\mathrm{mix}}$ of the solid solution and the formation of the solid solution. Ω represents the effect of the interaction of $\Delta S_{\mathrm{mix}}$ and $\Delta H_{\mathrm{mix}}$ on the phase formation. They believe that when Ω ≥ 1.1, δ ≤ 6.6%, the solid solution phase of HEAs will be stable. It can be pointed out in Table 3, that with the increase in Cr content, the δ, Δχ, and VEC of the AlCoCr*_x_*CuFe HECs decreased, while Ω increased. However, the $\Delta S_{\mathrm{mix}}$ value was not in direct or inverse proportion with the Cr content. The parameters of $\Delta S_{\mathrm{mix}}$, $\Delta H_{\mathrm{mix}}$, δ, Δχ, and Ω for the Cr05, Cr10, Cr15, and Cr20 HECs were consistent with previous studies. The VEC for the AlCoCr*_x_*CuFe HECs in this study were all between 6.87 and 8.6. The XRD results showed that AlCoCr*_x_*CuFe HECs had a FCC and BCC two-phase solid solution, which was in line with the results of related research [31]. The above-mentioned parameters were often used to predict the phase formation of fused and cast HEAs blocks. Laser cladding is characterized by concentrated heat, fast heating, fast cooling, and a small heat affected zone. The special heating and cooling process of AlCoCr*_x_*CuFe HECs may force its phase formation to be different from that of the block HEAs. However, the relationship between the phase composition of AlCoCr*_x_*CuFe HECs in this study and the parameters of $\Delta S_{\mathrm{mix}}$,$\;\Delta H_{\mathrm{mix}}$, *δ*, Δχ, VEC, and Ω were in good agreement with the phase formation range in existing research [31,32,33].

### 3.2. Microstructure Characterization

**Figure 3:**
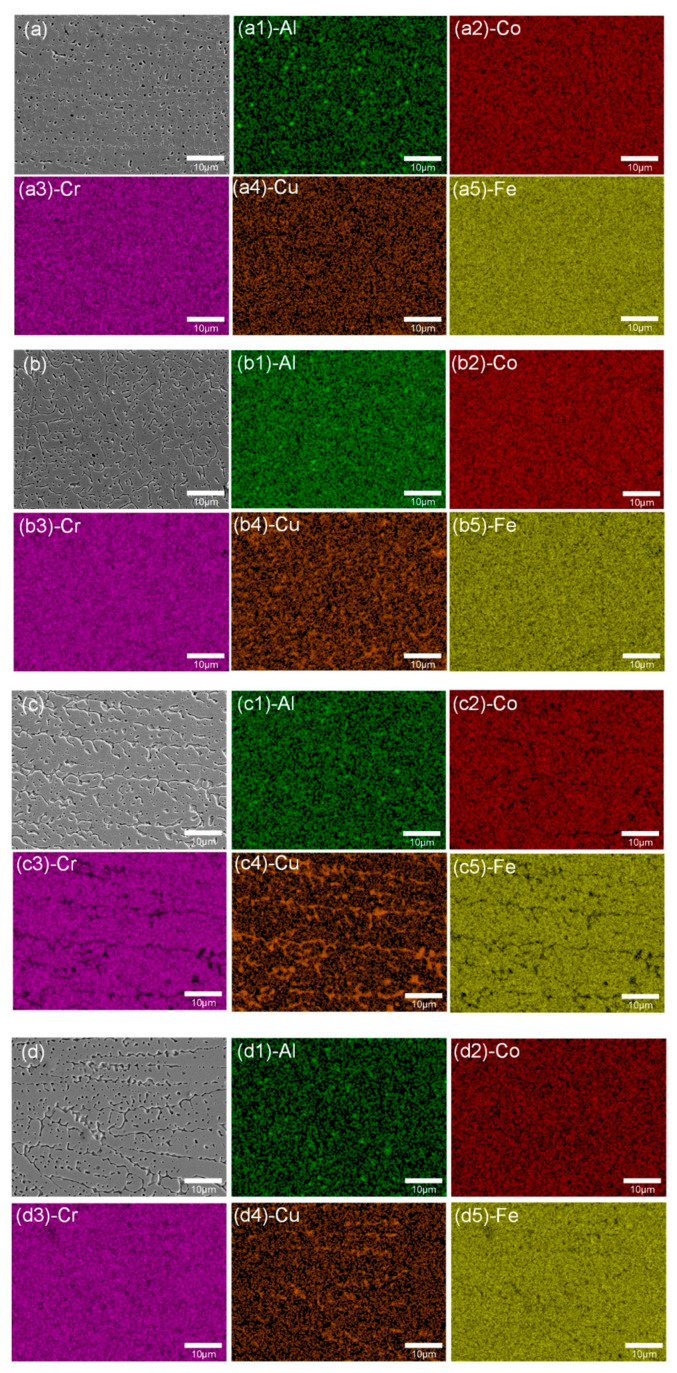
Microstructure morphologies of Cr05 (**a**), Cr10 (**b**), Cr15 (**c**), Cr20 (**d**) and EDS elemental mapping of Cr05-Al (**a1**), Cr05-Co (**a2**), Cr05-Cr (**a3**), Cr05-Cu (**a4**), Cr05-Fe (**a5**), Cr10-Al (**b1**), Cr10-Co (**b2**), Cr10-Cr (**b3**), Cr10-Cu (**b4**), Cr10-Fe (**b5**), Cr15-Al (**c1**), Cr15-Co (**c2**), Cr15-Cr (**c3**), Cr15-Cu (**c4**), Cr15-Fe (**c5**), Cr20-Al (**d1**), Cr20-Co (**d2**), Cr20-Cr (**d3**), Cr20-Cu (**d4**), Cr20-Fe (**d5**).

As shown in Figure 3, the microstructure of HECs was composed of dendrite (DR) structures and interdendrite (ID) structures. Laser cladding technology has the characteristics of high cooling rate (10^6^ K/s), and the solidification process of molten pool is non-equilibrium solidification [34]. The atoms do not have enough time for complete diffusion and composition homogenization, which makes the microstructure of the cladding layer show a dendrite structure [35]. Combined with EDS mapping and XRD results, the DR structures of the BCC phase were rich in Al, Fe, Cr, and Co elements, while Cu was concentrated in ID to form a copper-rich FCC structure. The enthalpy of mixing between Al and Co was very negative (Table 4), which made them well mutually soluble. Cr and Fe were little different in atomic radius and similar properties, therefore, they were mainly concentrated in the DR region to form BCC phases. A large number of Cu elements were enriched between the ID region because of the high enthalpy of mixing between Cu and other elements, which prevented Cu from existing in the DR region. Moreover, the poor binding ability with other elements of Cu led to its segregation in the ID region during solidification. In addition, with the increase in Cr content, the peak of BCC decreased, indicating that the content of BCC decreased accordingly.

### 3.3. Cavitation Erosion Performance and Mechanism

**Figure 4:**
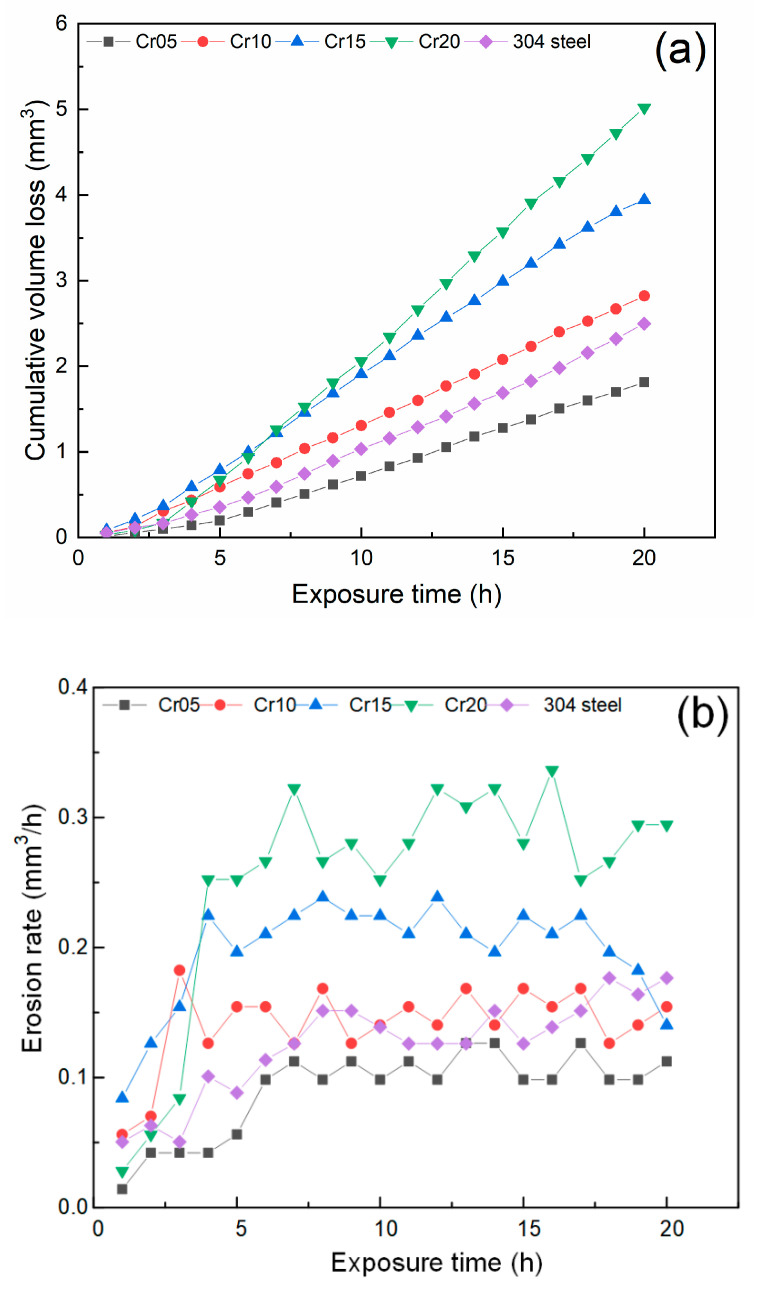
(**a**) Cumulative volume loss; (**b**) erosion rate of AlCoCr*_x_*CuFe HECs with the 20 h cavitation erosion test.

The curve in Figure 4a exhibits the cumulative volume loss of test samples during the 20 h cavitation erosion test. Volume loss (VL) was used to characterize the cavitation resistance of HECs.

It can be clearly seen that the change in the curve can be divided into three different cavitation erosion periods [7]: one is the incubation period with little material loss, the other is the period with severe material loss; and another stationary period with stable material loss. Cr05 HEC exhibited the lowest cumulative volume loss in all samples. With the increase in Cr content, the 20 h cumulative volume loss increased. This indicated that the cavitation erosion resistance of AlCoCr*_x_*CuFe HECs was decreased with the addition of Cr content, reaching its worst at Cr20 HEC. Figure 4b shows the erosion rate curves of the AlCoCr*_x_*CuFe HECs. The incubation period of Cr05 HEC was about 4 h. However, Cr10, Cr15, and Cr20 HECs only experienced about 2 h of the incubation period. Unlike from the stable growth of Cr05 HEC and AISI 304 steel in the accumulation period, the growth of Cr10 and Cr15 was very rapid, and they only experienced about 1 h of rapid growth. With the increase in Cr content, the erosion rate of HECs during the cavitation stationary period increased, which was consistent with their 20 h cavitation cumulative volume loss results.

**Figure 5:**
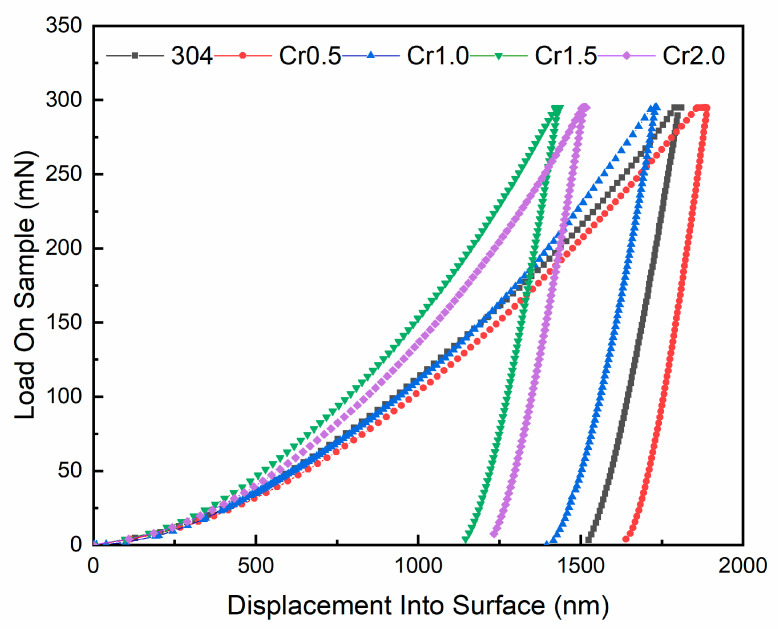
Load-displacement curves in nanoindentation test for AlCoCr*_x_*CuFe HECs and AISI 304 steel.

Figure 5 shows the nanoindentation characteristics of the AlCoCr_x_CuFe HECs and AISI 304 steel. The Oliver-Pharr method was used [36] for calculating the nanoindentation hardness and reduced elastic modulus of AlCoCr*_x_*CuFe HECs. The equations used for calculation were (8)–(10).

(8) $$h_{c}=h_{\max}-\varepsilon \frac{P_{\max}}{S}$$

(9) $$S=\frac{dP}{dh}$$

(10) $$A_{c}=24.5{\left(h_{c}+12.3\right)}^{2}$$

(11) $$H_{n}=\frac{P_{\max}}{A_{c}}$$

(12) $$E_{r}=\frac{S\sqrt{\pi }}{2\sqrt{A_{c}}}$$

where $h_{c}$, $h_{\max}$, ε, S,$\;A_{c}$, P, h,$\;H_{n}$, $E_{r}$ represent the contact depth, displacement with maximum load, correction constant (ε=0.75), unloading stiffness with maximum load, contact area, applied load in the process of nanoindentation, indentation depth, nanoindentation hardness, and reduced elastic modulus, respectively.

**Figure 6:**
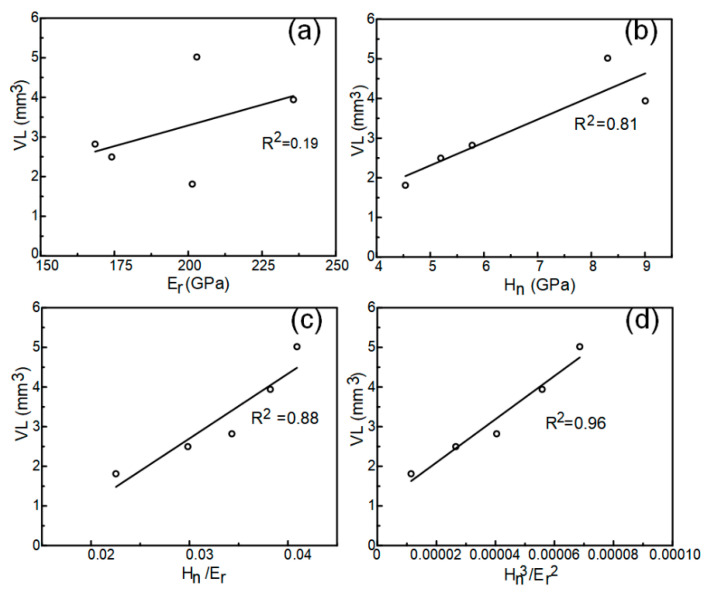
Correlation between the cumulative volume loss (VL) and (**a**) reduced elastic modulus $E_{r}$, (**b**) nanoindentation hardness $H_{n}$, (**c**) $H_{n}/E_{r}$, and (**d**) $H_{n}{}^{3}$/$E_{r}{}^{2}$.

The research of H. Attar et al. [37] indicated that the reduced elastic modulus of the nano indentation test was load dependent. In this study, the maximum load of the nano-indentation test was selected as 300 mN, aiming to make the indentation depth and cavitation erosion depth in the same order of magnitude. Hardness and modulus of elasticity are often regarded as mechanical parameters related to the cavitation resistance of a material [2]. The micro-mechanical performance parameters, drawn from the nano-indentation tests, are shown in Table 6. Figure 6 shows the relationship between $H_{n}$, $E_{r}$, $H_{n}/E_{r}$,$\;H_{n}{}^{3}$/$E_{r}{}^{2},$ and cumulative volume loss, respectively. The $E_{r}$ and VL correlation coefficient was only *R*^2^ = 0.19,$\;H_{n}$ and the VL correlation coefficient was *R*^2^ = 0.81. This indicates that $E_{r}$ and $H_{n}$ cannot affect the cavitation performance of HECs alone. $H_{n}/E_{r}$ is described as the elastic strain to failure [38] and is widely considered as a valuable measure to determine the elastic behavior limit of the contact surface. The $H_{n}/E_{r}$ and VL correlation coefficient was *R*^2^ = 0.88. This indicates that the proper combination of hardness and elasticity is an important reason for the improvement of cavitation erosion performance. However, an over high $H_{n}/E_{r}$ will weaken the shear strength and reduce the wear resistance [39]. Another parameter, $H_{n}{}^{3}$/$E_{r}{}^{2}$, was more related to VL (*R*^2^ = 0.96). A higher $H_{n}{}^{3}$/$E_{r}{}^{2}$ usually corresponds to a higher resistance to plastic deformation [40]. This indicates that the appropriate plastic deformation resistance was sufficient to bear the cavitation impacts. Cr05 HEC obtained an excellent combination of hardness and elasticity, which made a great contribution to the improvement in cavitation erosion resistance.

**Figure 7:**
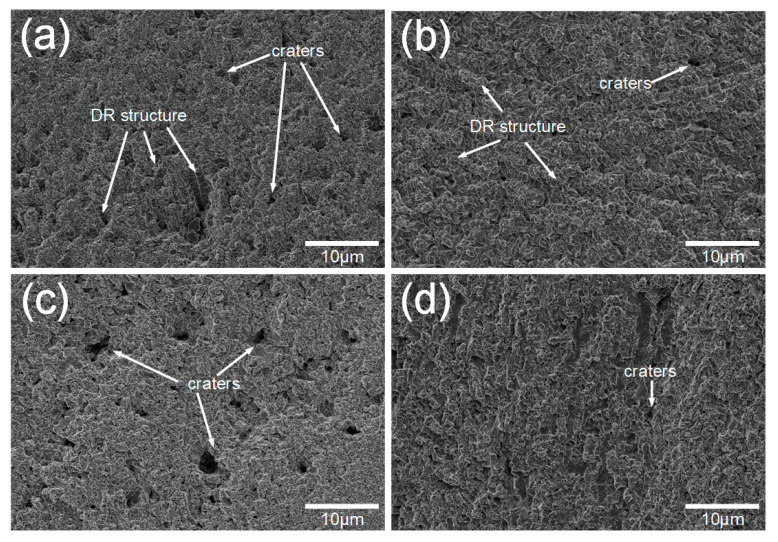
Surfaces of the AlCoCr*_x_*CuFe HECs with the 20 h cavitation erosion test: (**a**) Cr05 HEC; (**b**) Cr10 HEC; (**c**) Cr15 HEC; (**d**) Cr20 HEC.

**Figure 8:**
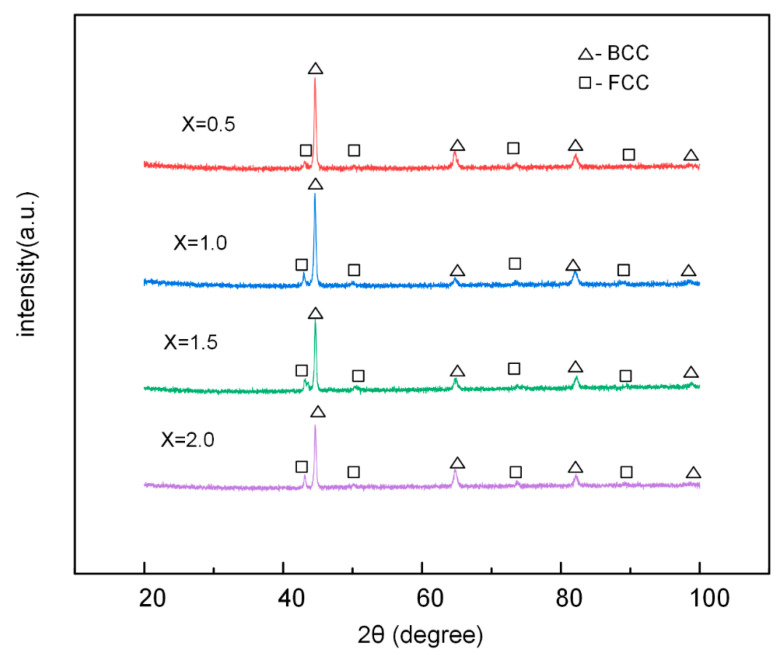
XRD patterns of the AlCoCr*_x_*CuFe HECs after the 20 h cavitation erosion tests.

Figure 7 exhibits the surfaces of the AlCoCr*_x_*CuFe HECs with the 20 h cavitation erosion test. The DR structure can be clearly observed on the surface of Cr05 and Cr10, where it can be inferred that the DR structure is more difficult to damage in the cavitation erosion test. The BCC phase of the DR structure can make it work hardening when it is impacted by cavitation erosion, which can prevent further damage to the material caused by cavitation impact [41]. FCC structures rich in copper tend to have a higher stacking fault energy [42]. Higher stacking fault energy can result in poor cavitation resistance [43], which makes the ID structure more vulnerable to cavitation impact. The relatively undamaged DR structure in Cr05 proves this point. Figure 8 shows the XRD pattern of the AlCoCr*_x_*CuFe HECs after the 20 h cavitation erosion test. Compared with the XRD patterns of HECs with different Cr contents before the cavitation erosion test, it can be found that the characteristic peak of the FCC phase decreased significantly near 50°, especially in Cr05 HEC. This also coincides with the surface morphology after the cavitation erosion test in Figure 7. The exposed DR structure was caused by the damage of the ID structure by cavitation impact.

**Figure 9:**
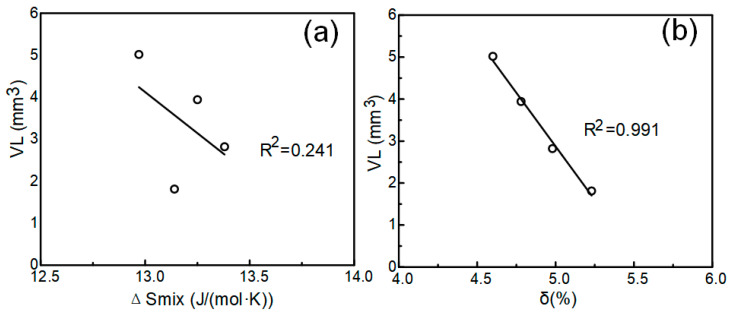
Correlation between the cumulative volume loss (VL) and (**a**) mixed entropy $\Delta S_{\mathrm{mix}}$, (**b**) atomic radius difference δ.

The parameters $\Delta S_{\mathrm{mix}}$, and δ influence the properties of high entropy alloys by influencing the phase formation process, so it is necessary to discuss the relationship between these parameters and the cavitation erosion resistance (expressed in VL). As shown in Figure 9, there was no obvious correlation between $\Delta S_{\mathrm{mix}}$ and VL (correlation coefficient *R*^2^ = 0.241), while there was a strong correlation between δ and VL (correlation coefficient *R*^2^ = 0.991). The parameter $\Delta S_{\mathrm{mix}}$ can represent the driving force of the formation of the solid solution [44]. High $\Delta S_{\mathrm{mix}}$ will increase the degree of entropy chaos in the alloy system and significantly reduce the free energy of the alloy. The disordered distribution of different alloying elements in the crystal lattice promotes the formation of a solid solution. However, in Figure 9a, VL and $\Delta S_{\mathrm{mix}}$ did not show a strong correlation. At the same time, δ showed a strong correlation with VL. The collapse of cavitation in water can produce high temperatures of 2300 k–5100 k [45]. The heat conduction in solid is realized by phonons and free electrons. With the decrease in Cr content, the difference in atomic radius increases. The serious lattice distortion of high entropy alloys leads to the scattering of phonons and lattice, which reduces the thermal conductivity [46]. This also makes the high entropy alloy have certain advantages in the face of cavitation heat.

## 4. Conclusions

(1) The AlCoCr*_x_*CuFe HECs with different Cr contents were FCC and BCC duplex solid solution phase. With the increase in Cr content, the FCC phase content increased gradually. The phase formation of HECs were the result of competition among $\Delta S_{\mathrm{mix}}$,$\;\Delta H_{\mathrm{mix}}$, δ, Δχ, VEC, and Ω.

(2) The microstructure of the AlCoCr*_x_*CuFe HECs was dendrite in the BCC phase and interdendrite structures in the FCC phase. With the increase in Cr content, copper segregation occurred in dendritic regions, especially in the Cr15 and Cr20 HECs.

(3) Cavitation erosion resistance expressed by 20 h volume loss was decreased with the addition of the Cr element. Cr05 HEC revealed the best cavitation erosion resistance among all samples. The surface after 20 h cavitation erosion tests exposed the dendritic structure of the BCC phase, which was caused by the destruction of ID structure by the cavitation impact. The cavitation resistance of the AlCoCr*_x_*CuFe HECs had good correlation with the mechanical parameter $H_{n}{}^{3}$/$E_{r}{}^{2}$ and phase formation parameter δ. The cavitation corrosion resistance of Cr05 was significantly better than that of AISI 304 steel, which provides a new possibility for the preparation of cavitation erosion resistant coatings by laser cladding.

## Figures and Tables

**Table 1:** The nominal composition of AISI 304 steel used in current study.

C	Si	Cr	Mn	Ni	P	S	Fe
0.08	1.0	19.0	2.0	11.0	0.035	0.03	bal.

**Table 2:** Laser cladding parameters used in current study.

Laser Power	Powder Feeding Speed	Scanning Speed	Spot Diameter	Shielding Gas and Its Flow Rate	Overlapping Ratio
1400 w	20 g/min	350 mm/min	4 mm	Ar 3.5 NL/min	50%

**Table 3:** Parameters of $\Delta S_{\mathrm{mix}}$, $\Delta H_{\mathrm{mix}}$, δ, Δχ, VEC, and Ω for the AlCoCr*_x_*CuFe HECs (high entropy alloy coatings).

HECs	$\Delta S_{\mathrm{mix}}\;J/(\mathrm{mol}\cdot K)$	$\Delta H_{\mathrm{mix}}\;\mathrm{kJ}/\mathrm{mol}$	δ	Δχ	VEC	Ω
Cr05	13.14	−2.86	5.23%	0.118683	7.55	5.82
Cr10	13.38	−2.56	4.98%	0.118423	7.40	6.93
Cr15	13.25	−2.31	4.78%	0.118199	7.27	7.88
Cr20	12.97	−2.11	4.60%	0.118005	7.16	8.69

**Table 4:** Mixing enthalpies between the elements used in the current study [30].

$\Delta H_{\mathrm{mix}}\;\mathrm{kJ}/\mathrm{mol}$	AL	Co	Cr	Fe	Cu
Al	-	−19	−10	−11	−1
Co	-	-	−4	−1	6
Cr	-	-	-	−1	12
Fe	-	-	-	-	13
Cu	-	-	-	-	-

**Table 5:** The atomic radius, electronegativity, and VEC used in the current study.

	Al	Co	Cr	Fe	Cu
Electronegativity	1.61	1.88	1.66	1.83	1.9
Atomic Radius (nm)	0.143	0.125	0.128	0.127	0.128
VEC	3	9	6	8	11

**Table 6:** Various mechanical parameters of the AlCoCr*_x_*CuFe HECs.

HEC	$E_{r}\;(\mathrm{GPa})$	$H_{n}\;(\mathrm{GPa})$	$H_{n}$ $/E_{r}$	$H_{n}{}^{3}$ $/E_{r}{}^{2}$
AISI 304	174.1 ± 9.6	5.19 ± 0.14	0.0298	0.00002658
Cr05	201.4 ± 6.4	4.54 ± 0.21	0.0225	0.00001143
Cr10	168.4 ± 6.1	5.78 ± 0.13	0.0343	0.00004040
Cr15	235.6 ± 12.7	9.01 ± 0.44	0.0382	0.00005582
Cr20	202.8 ± 8.5	8.30 ± 0.17	0.0409	0.00006856
